# Trimethylamine N‐Oxide Linking Gut Microbiota to Cardiocerebral Disease Is a Novel Biomarker for Stroke Subtypes With Mixed Etiology: A Prospective Cohort Study

**DOI:** 10.1002/cns.70704

**Published:** 2025-12-15

**Authors:** Yukun Wang, Yeju Hu, Ruoyu Qin, Wei Li, Chu Zhou, Rongrong Liu, Qiyang Yuan, Ruicheng Zhang, Guiyun Cui, Shiguang Zhu

**Affiliations:** ^1^ Department of Neurology, the Affiliated Hospital of Xuzhou Medical University Xuzhou China; ^2^ The First Clinical College Xuzhou Medical University Xuzhou China; ^3^ Jiangsu Province Key Laboratory of Anesthesiology Xuzhou Medical University Xuzhou China

**Keywords:** cardiocerebral disease, etiology, gut, microbiota, stroke, trimethylamine N‐oxide

## Abstract

**Background:**

The pathogenesis of ischemic stroke is multifaceted, and growing evidence highlights that mixed etiologies should be considered. This prospective cohort study investigated the relationship between plasma levels of trimethylamine N‐oxide (TMAO), a cardiovascular disease risk factor, and etiologic stroke subtypes.

**Methods:**

Plasma TMAO levels were compared in 223 patients, including 95, 73, and 55 patients with large artery atherosclerosis (LAA), cardioembolism (CE), and cardioembolism with culprit artery stenosis (CES), respectively, admitted with acute ischemic stroke complicated by large vessel occlusion and treated with endovascular therapy. At‐admission clinical data and blood samples obtained during intervention were collected.

**Results:**

After adjusting for covariates, including age, sex, hypertension, diabetes mellitus, estimated glomerular filtration rate(eGFR), smoking, and alcohol consumption, plasma TMAO levels were highest in the CES group (1.583 μmol/L), followed by the LAA and CE groups (1.064 and 0.583 μmol/L, respectively), with significant differences among the three groups detected (Wald χ^2^ = 22.877, *p* < 0.001). By binary logistic regression analysis after adjusting for the same covariates, plasma TMAO level was an independent predictor for distinguishing the CES subtype from the CE subtype (95% CI, 2.062–8.183; *p* < 0.001), with an area under the curve of 0.778.

**Conclusion:**

Plasma TMAO levels, which were highest in patients with stroke due to CES, followed by those with stroke due to LAA and CE, may serve as an independent predictor for distinguishing CE from CES as the stroke etiology.

## Graphical abstract

After the human body ingests foods rich in choline, betaine, and L‐carnitine, the intestinal microbiota can metabolize these dietary nutrients to form trimethylamine (TMA) through the diet. TMA then enters the liver through the portal vein and is subsequently oxidized to trimethylamine N‐oxide (TMAO) in the presence of flavin‐containing monooxygenases 3 (FMO3). Multiple studies have shown that changes in TMAO levels can predict the risk of stroke in patients with ischemic stroke, as well as the severity of the stroke, clinical outcomes, and mortality.

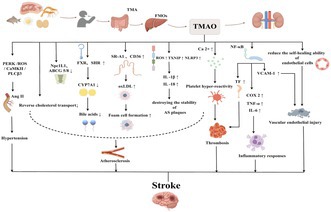

## 1 Introduction

Accumulating evidence reveals the association of intestinal dysbiosis, caused by alterations in gut microbiota diversity and abundance, with the onset, progression, and clinical outcomes of ischemic stroke [1]. During digestion, gut microbiota play an important role in metabolizing dietary nutrients, such as choline, betaine, and L‐carnitine, into trimethylamine, which then enters the liver via the portal vein, where it is oxidized into trimethylamine N‐oxide (TMAO) by flavin‐containing monooxygenase 3 [2]. Multiple studies have indicated that fluctuations in TMAO levels can predict the risk [3], severity [4, 5], clinical outcomes and mortality [6] of ischemic stroke. However, the relationship between TMAO levels and ischemic stroke with specific etiologies remains unclear.

Recent studies indicate that TMAO may contribute to the development and progression of ischemic stroke through several mechanisms, including the impairment of vascular endothelial function, promotion of foam cell formation, disruption of cholesterol metabolism, and increased platelet reactivity [7, 8, 9, 10]. TMAO is recognized as a proatherogenic molecule [11] associated with cardiovascular disease [12], which shares risk factors and pathophysiologic mechanisms with ischemic stroke related to large artery atherosclerosis (LAA) [13]. Furthermore, recent studies have identified TMAO as an independent risk factor for ischemic stroke in patients with atrial fibrillation (AF) [14]. TMAO has been shown to be associated with increased platelet hyperreactivity and thrombosis risk [15]. Higher circulating levels of TMAO, betaine, and choline are accompanied by increased platelet reactivity in patients with AF who develop thrombosis [16]. Moreover, atherosclerotic thrombosis and thromboembolism often coexist in patients with AF [17], with studies confirming the association between TMAO levels and cerebrovascular lesions in these individuals [18]. It is reasonable to assume that plasma TMAO levels differ among patients with LAA, cardioembolism (CE), and cardioembolism with culprit artery stenosis (CES) who experience stroke and that TMAO serves as an independent predictor that can distinguish the etiology of stroke. To test this hypothesis, we investigated the relationship between plasma TMAO levels and specific subtypes of stroke.

## 2 Methods

### 2.1 Study Design and Patient Selection

This prospective cohort study included patients who presented with acute ischemic stroke due to large vessel occlusion and underwent endovascular therapy at Stroke Alliance of the Affiliated Hospital of Xuzhou Medical University between December 10, 2023 and December 31, 2024. Patients aged ≥ 18 years with a confirmed diagnosis of acute ischemic stroke with large vessel occlusion affecting the internal carotid, anterior cerebral, middle cerebral, vertebral, or basilar artery, verified by computed tomography angiography or digital subtraction angiography (DSA) were included. The exclusion criteria were as follows: [1] other cerebrovascular diseases, such as intracranial hemorrhage, transient ischemic attack, cerebral aneurysm, and vascular malformation; [2] stroke etiology other than LAA, CE, or CES; [3] severe systemic disease, such as cancer, infections, and inflammatory diseases; [4] prior use of probiotics or antibiotics within 1 month before admission; [5] concurrent participation in any clinical trial; and [6] severe comorbidities, such as congestive heart or respiratory failure and renal or hepatic insufficiency.

### 2.2 Baseline Data Collection

Baseline demographic and clinical data, including age, sex, estimated glomerular filtration rate(eGFR), smoking history, alcohol consumption history, and relevant medical history, such as ischemic stroke, AF, hypertension, and diabetes mellitus, were systematically obtained by trained neurologists. Stroke severity at admission was assessed using the National Institutes of Health Stroke Scale (NIHSS), and functional outcome at discharge was evaluated using the modified Rankin scale. To minimize bias, both the patients and investigators involved in clinical evaluation were blinded to the results of the biochemical tests.

### 2.3 Stroke Etiology and Subtyping

Stroke etiology was classified as CE, LAA, and CES, based on the TOAST (Trial of Org 10,172 in Acute Stroke Treatment) classification criteria and the protocol utilized in the DIRECT‐MT trial, by experienced neurologists. Stroke due to cardioembolism was defined as a documented history of AF or mechanical aortic or mitral valve replacement, verified by an electronic data capture system, in the absence of evidence of concurrent intracranial atherosclerosis or extracranial internal carotid artery pathology, such as moderate‐to‐severe stenosis (50%–99%), occlusion (100%), and dissection. Stroke due to LAA was defined as the confirmation of atherosclerotic stenosis of ≥ 50% in the culprit artery by DSA or as the presence of secondary occlusion attributable to atherosclerosis in the ipsilateral extracranial internal carotid artery in the absence of identifiable high‐risk sources of cardioembolism. Stroke due to CES was determined based on two criteria: [1] residual atherosclerotic stenosis of > 50% in the culprit artery after thrombectomy, confirmed by DSA, with the exclusion of dissection or vasculitis and [2] the presence of concurrent AF or significant valvular heart disease.

### 2.4 Measurement of Plasma TMAO Levels

During percutaneous intracranial thrombectomy conducted by certified neurointerventionalists, a microcatheter was advanced over a microwire to the occlusion site. Following the deployment of a stent retriever or positioning of an aspiration catheter, a 5‐mL arterial blood sample was aspirated proximal to the thrombus using an external syringe connected to the microcatheter. Blood samples were collected into serum separator and ethylenediaminetetraacetic acid‐containing tubes. The collected samples were centrifuged at 2000 rpm for 10 min to isolate plasma, which was immediately aliquoted and stored at −80°C until analysis. All processing steps, including sample separation, aliquoting, and transport, were performed under cryogenic conditions.

For TMAO extraction, an appropriate amount of plasma was transferred into a 2‐mL centrifuge tube, and 10 μL of internal standard solution was accurately added, followed by 750 μL of 1% formic acid‐acetonitrile solution. The mixture was vortexed for 30 s using a multitube vortex mixer and centrifuged at 12,000 rpm and 4°C for 5 min using a high‐speed bench refrigerated centrifuge. Then, 500 μL of the supernatant was passed through a 0.22‐μm filter membrane and transferred to an LC–MS vial. Plasma TMAO levels were quantified using high‐performance liquid chromatography coupled with tandem mass spectrometry (LC–MS/MS). Quality control (QC) samples were prepared by pooling supernatant from study samples and mixing with the cal5 calibration standard at a 1:1 volume ratio. QC samples were analyzed only when the total number of study samples exceeded 10; no QC analysis was performed for sample sets of fewer than 11. A single laboratory technician blinded to all clinical data performed the assay.

### 2.5 Statistical Analysis

Categorical variables were presented as proportions, whereas continuous variables were presented as medians with interquartile ranges (IQRs) or means with standard deviation. The Kolmogorov–Smirnov test was employed to assess the normality of data distribution. The χ [2] test was utilized for categorical variables. The Kruskal–Wallis test was used to compare the distribution of plasma TMAO levels among the LAA, CE, and CES groups. Baseline clinical characteristics and selected confounding factors were adjusted, and the Kolmogorov–Smirnov test was used to confirm the gamma distribution of plasma TMAO levels. A gamma generalized linear model was used to analyze the relationship between plasma TMAO levels and stroke subtypes, with sequential Bonferroni correction applied for pairwise comparisons of differences between groups. In subsequent binary logistic regression analysis, baseline clinical characteristics and other selected confounding factors were adjusted to evaluate plasma TMAO level as an independent predictor of stroke due to CE or CES. A two‐sided *p* < 0.05 was considered statistically significant. All analyses were performed using SPSS (version 27; IBM, Armonk, NY, USA).

## 3 Results

### 3.1 Baseline Characteristics of the Study Participants

**FIGURE 1:**
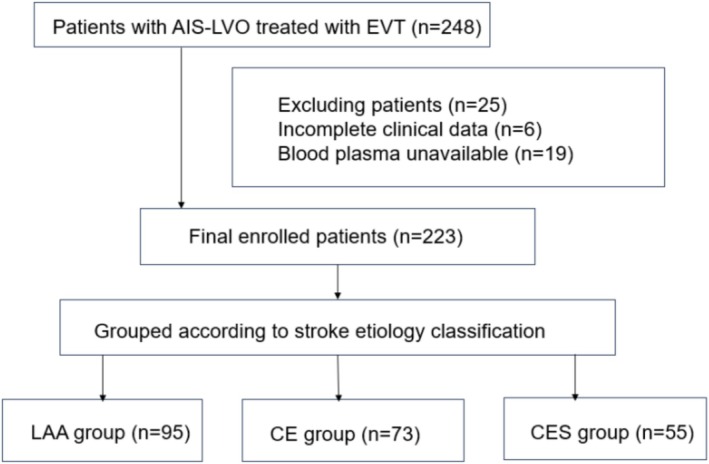
Study flowchart. AIS‐LVO, Acute Ischemic Stroke due to Large Vessel Occlusion; EVT, Endovascular Thrombectomy; CE, cardioembolism; CES, cardioembolism with culprit artery stenosis; LAA, large artery atherosclerosis.

Figure 1 depicts the flowchart of participant enrollment. Among a total of 248 patients who were initially enrolled, 19 patients with missing blood samples and 6 patients who did not fulfill the eligibility criteria were excluded; therefore, the final cohort included 223 patients.

The final cohort included 95, 73, and 55 patients with stroke due to LAA, CE, and CES, respectively. The overall cohort included 144 male patients (64.6%) with a median age of 68 years (IQR, 60–75 years) and a median baseline NIHSS score of 18 (IQR, 13–24.5).

The three stroke groups exhibited several significant differences (Table 1). The highest median age (74 [IQR, 67–79] years; *p* < 0.001), the most severe neurological deficits (median NIHSS score, 21 [IQR, 18–26]; *p* = 0.003), and the highest prevalence of AF (98.2%; *p* < 0.001) were observed in the CES group. In contrast, the highest rates of hypertension (72.6%; *p* < 0.001) and diabetes mellitus (35.8%; *p* = 0.007) and the largest proportion of males (73.7%; *p* = 0.005) were observed in the LAA group. Conversely, the CE group had significantly lower plasma TMAO levels (median, 0.54 [IQR 0.16–0.87] μmol/L; *p* < 0.001) and a milder stroke severity (median NIHSS score, 16 [IQR 12–21]; *p* = 0.003) compared with the other two groups.

**TABLE 1:** Baseline characteristics of patients categorized according to the stroke etiology.

Characteristics	TOTAL (*n* = 223)	LAA (*n* = 95)	CE (*n* = 73)	CES (*n* = 55)	*p*
Age (y)	68 (60–75)	65 (59–73)	67 (57–74)	74 (67–79)	< 0.001
Sex (male/female)	144/79	70/25	48/25	26/29	0.005
Baseline NIHSS score	18 (13–24.5)	18 (12–25)	16 (12–21)	21 (18–26)	0.003
Medical history					
Hypertension	121 (54.26%)	69 (72.63%)	19 (26.03%)	33 (60%)	< 0.001
Diabetes mellitus	57 (25.56%)	34 (35.79%)	11 (15.07%)	12 (21.82%)	0.007
Atrial fibrillation	122 (54.71%)	0 (0%)	68 (93.15%)	54 (98.18%)	< 0.001
Previous ischemic stroke	53 (23.77%)	21 (22.11%)	17 (23.29%)	15 (27.27%)	0.769
Smoking	31 (13.9%)	16 (16.84%)	7 (9.59%)	8 (14.55%)	0.4
Alcohol consumption	26 (11.66%)	11 (11.58%)	8 (10.96%)	7 (12.73%)	0.953
TMAO (μmol/L)	0.7 (0.34–1.45)	0.63 (0.34–1.41)	0.54 (0.16–0.87)	1.43 (0.6–2.37)	< 0.001
eGFR (mL/min per 1.73 m2)	99.81 (86.09–107.66)	101.99 (86–108.58)	101.76 (91.35–111.38)	92.69 (81.33–102.77)	0.004

*Note:* Continuous data are presented as medians (IQR) and categorical variables are presented as numbers (%).

Abbreviations: CE, cardioembolism; CES, cardioembolism with culprit artery stenosis; IQR, interquartile range; LAA, large artery atherosclerosis; NIHSS, National Institutes of Health stroke scale; TMAO, trimethylamine N‐oxide; eGFR, estimated glomerular filtration rate.

### 3.2 Comparison of Baseline Plasma TMAO Levels Among the Stroke Subtypes

**FIGURE 2:**
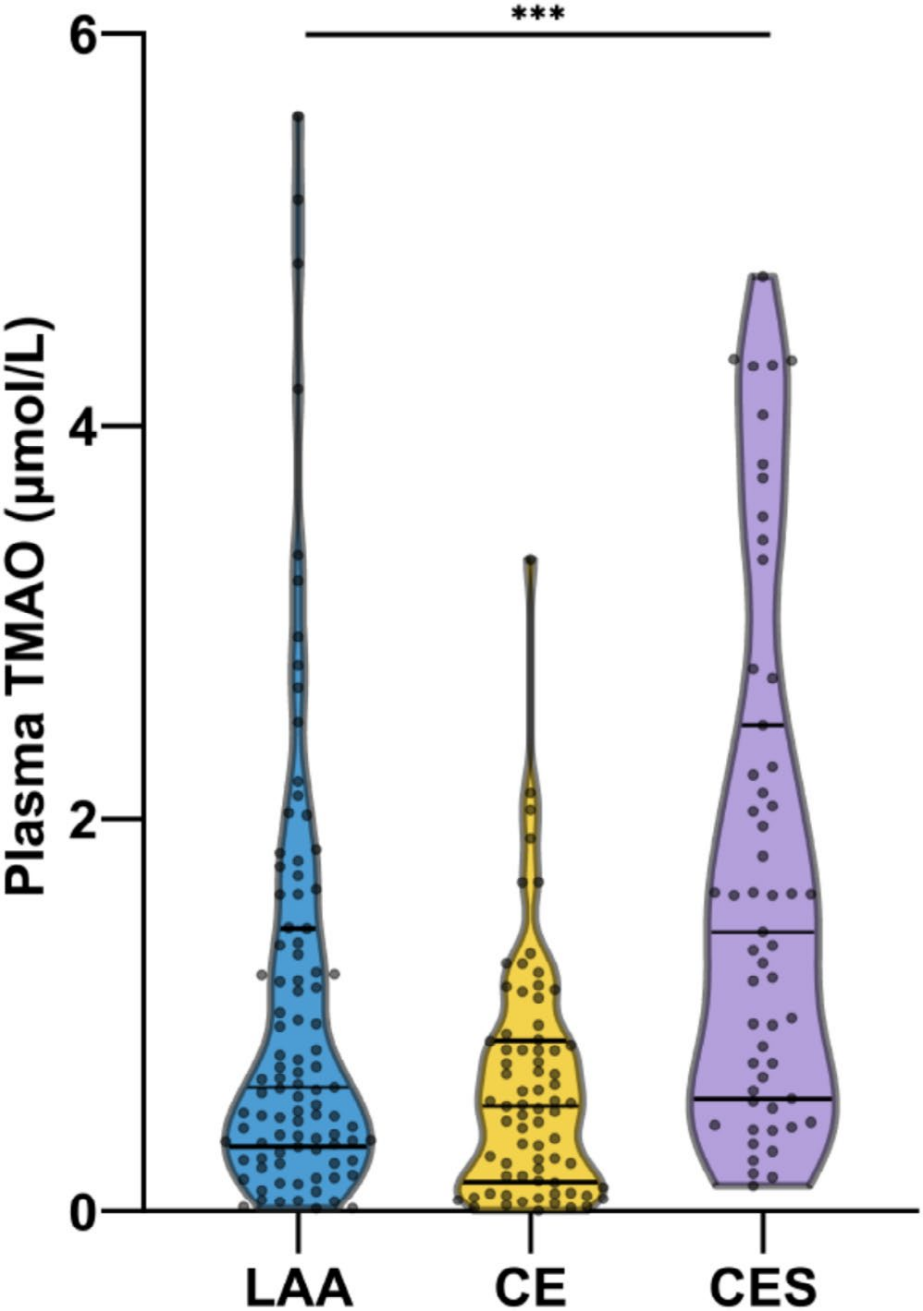
Plasma trimethylamine N‐oxide levels in study groups categorized based on stroke etiology. ***p < 0.001. CE, cardioembolism; CES, cardioembolism with culprit artery stenosis; LAA, large artery atherosclerosis; TMAO, trimethylamine N‐oxide.

As shown in Figure 2, plasma TMAO levels at admission significantly differed among the LAA, CE, and CES groups (0.63 [IQR, 0.34–1.41], 0.54 [IQR, 0.16–0.87], and 1.43 [IQR, 0.6–2.37] μmol/L, respectively; *p* < 0.001). Subsequently, we employed Gamma generalized linear modeling, which confirmed that plasma TMAO levels at admission were strongly associated with stroke subtypes ([2] Wald χ, 27.81; *p* < 0.001). Notably, even after adjusting for covariates, such as age, sex, hypertension, diabetes mellitus, eGFR, smoking, and alcohol consumption, the intergroup differences in plasma TMAO levels persisted (Wald χ2, 22.877; *p* < 0.001).

Using CES as the reference group, the geometric mean ratio of the LAA group was 0.672 (95% confidence interval [CI], 0.478–0.945; *p* = 0.022), indicating that the geometric mean TMAO was 1.49‐fold higher in patients with stroke due to CES compared to those with stroke due to LAA. Additionally, the geometric mean ratio of the CE group was 0.368 (95% CI, 0.257–0.528; *p* < 0.001), indicating that the geometric mean TMAO was 2.72‐fold higher in patients with stroke due to CES compared to those with stroke due to CE (Table 2).

**TABLE 2:** Mean ratios and mean difference of plasma TMAO levels between stroke subtypes.

Parameters	Unadjusted model		Adjusted model	
	Mean ratios/difference (95% CI)	*p*	Mean ratios/difference (95% CI)	*p*
Mean ratios				
LAA vs. CES	0.606 (0.440–0.835)	0.002	0.672 (0.478–0.945)	0.022
CE vs. CES	0.367 (0.262–0.515)	< 0.001	0.368 (0.257–0.528)	< 0.001
Mean difference				
LAA vs. CE	0.415 (0.131–0.699)	0.002	0.481 (0.158–0.803)	0.002
CES vs. LAA	0.685 (0.197–1.173)	0.006	0.519 (0.034–1.003)	0.036
CES vs. CE	1.1 (0.532–1.668)	< 0.001	0.999 (0.424–1.576)	< 0.001

*Note:* Using gamma generalized linear models log link function; Adjusted for age, sex, hypertension, Diabetes mellitus, smoking, and alcohol consumption.

Abbreviations: CI, confidence interval; eGFR, estimated glomerular filtration rate; TMAO, trimethylamine N‐oxide.

**FIGURE 3:**
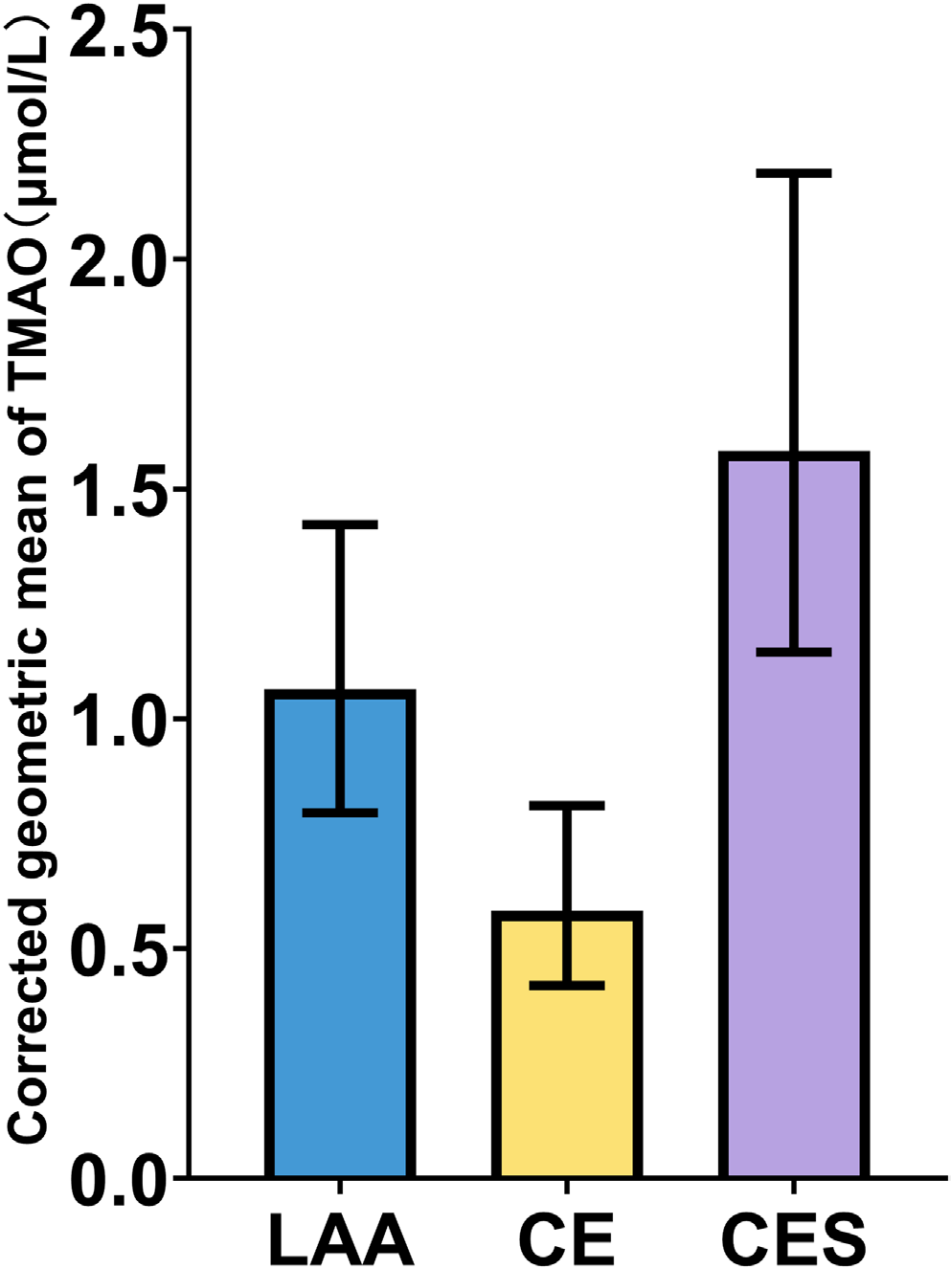
Corrected geometric mean plasma TMAO levels in groups categorized based on stroke etiology. CE, cardioembolism; CES, cardioembolism with culprit artery stenosis; LAA, large artery atherosclerosis; TMAO, trimethylamine N‐oxide.

Figure 3 shows the comparison of marginal mean TMAO levels derived from the gamma generalized linear model, following adjustment for age, sex, hypertension, diabetes mellitus, eGFR, smoking, and alcohol consumption. The mean plasma TMAO level was significantly higher in the CES group (1.583 [95% CI, 1.145–2.187] μmol/L) than in the LAA and CE groups (1.064 [95% CI, 0.795–1.423] and 0.583 [95% CI, 0.42–0.811] μmol/L).

Sequential Bonferroni‐corrected pairwise comparisons (Table 2) confirmed significant differences in plasma TMAO levels among the three groups after covariate adjustment. Specifically, plasma TMAO levels were significantly higher in the LAA group than in the CE group (mean difference, 0.481 [95% CI, 0.158–0.803] μmol/L; *p* = 0.002). Moreover, plasma TMAO levels were significantly higher in the CES group than in the LAA and CE groups (mean difference, 0.519 [95% CI, 0.034–1.003] μmol/L; *p* = 0.036 and 0.999 [95% CI, 0.424–1.576]; *p* < 0.001, respectively). These results revealed a clear gradient of plasma TMAO levels, with the highest levels observed in patients with stroke due to CES, followed by those with stroke due to LAA and those with stroke due to CE.

### 3.3 Association Between Elevated Plasma TMAO Levels and Stroke Due to CE and CES

**FIGURE 4:**
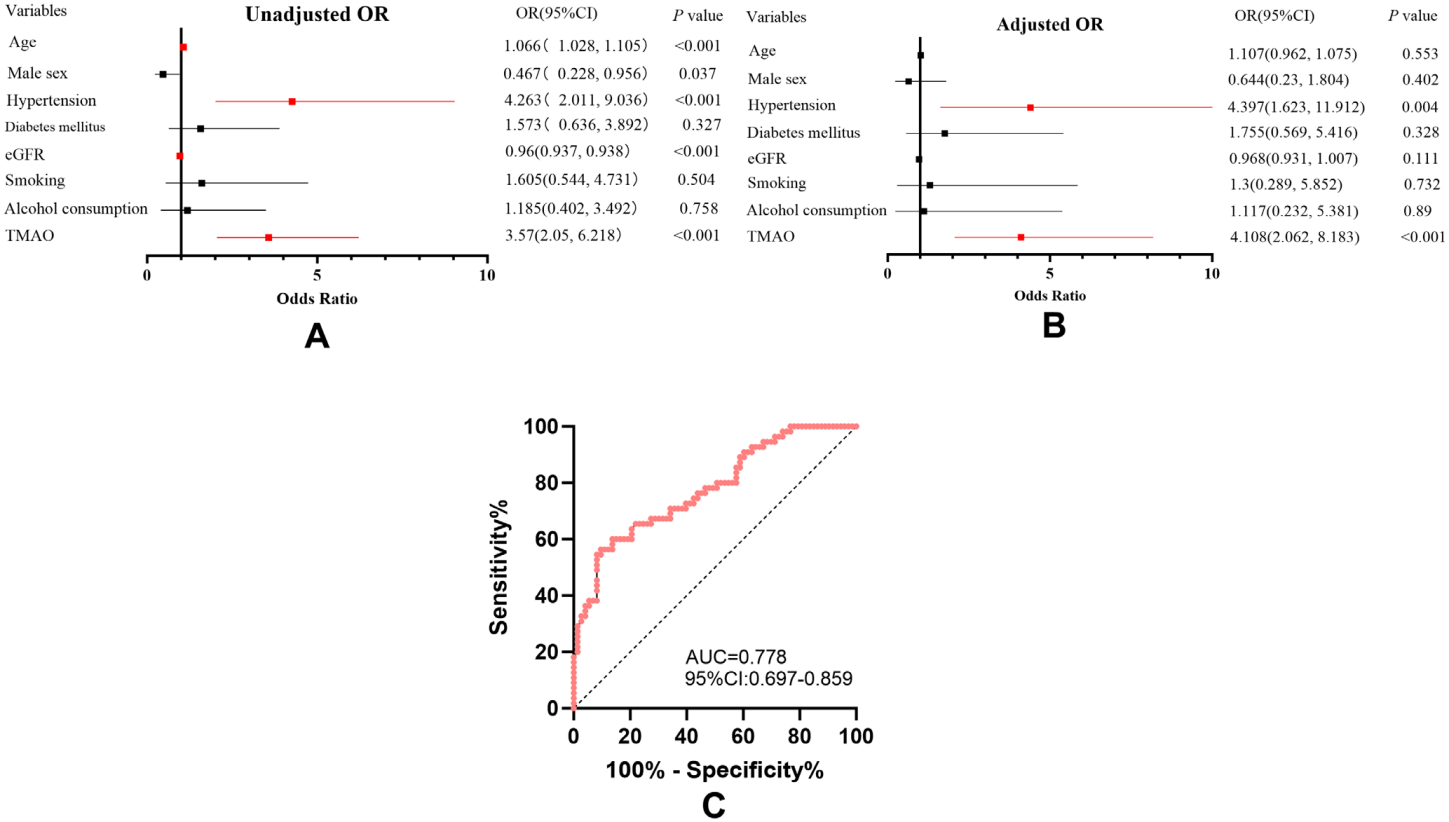
(A) Unadjusted logistic regression analyses of stroke etiology; (B) Adjusted logistic regression analyses of stroke etiology; (C) Receiver operator characteristic curve of plasma TMAO levels for discriminating between stroke due to cardioembolism and cardioembolism with culprit artery stenosis. OR, odds ratio; CI, confidence interval; Adjusted for age, sex, hypertension, Diabetes mellitus, eGFR, smoking, and alcohol consumption.

To ensure that TMAO levels are not influenced by clinical factors, it is necessary to adjust for confounding factors. We conducted a multiple regression analysis to eliminate confounding factors that might affect the association between TMAO and the etiological classification of stroke, such as risk factors for cerebrovascular diseases. By binary logistic regression analysis comparing the CE and CES groups, plasma TMAO level was an independent predictor of stroke due to CES (unadjusted odds ratio, 3.57 [95% CI, 2.05–6.22]; *p* < 0.001) (Figure 4A). The observed association persisted after adjusting for age, sex, hypertension, diabetes mellitus, eGFR, smoking, and alcohol consumption (adjusted odds ratio, 4.108 [95% CI, 2.062–8.183]; *p* < 0.001) (Figure 4B). Receiver operating characteristic analysis revealed that the plasma TMAO levels had a moderate discriminatory power in differentiating the CE and CES subtypes of stroke, resulting in an area under the curve of 0.778 (95% CI, 0.697–0.859; *p* < 0.001), with favorable sensitivity and specificity (Figure 4C).

## 4 Discussion

In the present study investigating plasma TMAO levels across three etiologic subtypes of acute ischemic stroke due to large vessel occlusion, our analyses revealed that plasma TMAO levels at admission were significantly lower in the patients with stroke due to LAA as well as in those with stroke due to CE, compared with the patients with stroke due to CES, with the observed difference persisting after multivariable adjustment by gamma generalized linear modeling. Further analyses established a clear gradient of plasma TMAO levels, with the highest levels observed in patients with stroke due to CES, followed by those with stroke due to LAA and those with stroke due to CE. Moreover, binary logistic regression confirmed plasma TMAO level as an independent predictor for distinguishing stroke due to CE from stroke due to CES.

Current evidence linking plasma TMAO levels to stroke pathogenesis remains limited. While most studies report elevated TMAO levels in association with increased stroke risk and severity as well as with poor functional outcomes, ischemic stroke is a heterogeneous condition with diverse underlying mechanisms. Biologic pathways connecting TMAO to specific stroke etiologies remain unclear [19, 20]. In this study, blood samples were collected from the responsible artery using a microcatheter to quantitatively measure TMAO and its precursor levels. This approach avoided potential interferences, thereby enhancing the specificity and reliability of the study result. The observed pattern suggests that stroke due to CES represents a distinct entity with mixed etiology beyond that considered based on traditional classifications.

The lower plasma TMAO levels measured in patients with stroke due to LAA and in those with CE, compared to those with stroke due to CES, indicate that distinct pathophysiologic mechanisms may be at play. In LAA, TMAO promotes atherosclerosis through endothelial dysfunction [21, 22, 23], disrupted cholesterol metabolism [24, 25], vascular smooth muscle cell inflammation [26, 27] and plaque destabilization [28, 29, 30]. The lower plasma TMAO levels found in the LAA group compared with the CES group indicate that intracranial atherosclerosis alone may not be sufficient in increasing plasma TMAO levels. Instead, synergistic interactions between cardiac factors, such as endothelial injury due to AF, and vascular pathology, potentially mediated by gut‐derived metabolites such as TMAO, may trigger a distinct pathologic cascade.

In pure cardioembolic events, where thrombi arise from cardiac sources, lower plasma TMAO levels suggest that embolism mechanisms are less reliant on TMAO‐associated atherosclerotic pathways and are more likely to be impacted by cardiac hemodynamics and endothelial changes. TMAO contributes to the promotion of AF through several mechanisms, including fibrosis [31], electrical remodeling [32] and structural alterations [33]. Additionally, TMAO enhances platelet reactivity and thrombosis [15, 34]. Furthermore, vascular endothelial tissue factor is involved in facilitating TMAO‐mediated arterial thrombogenesis [35, 36]. Elevated plasma TMAO levels have been identified as an independent predictor of stroke in patients with AF, [14] with increased levels particularly noted in those with AF and thrombosis [16].

In the present study, the elevated plasma TMAO levels observed in patients with stroke due to CES likely reflect the presence of AF and atherosclerotic stenosis, two pathologic mechanisms at play in these individuals. Within this framework, TMAO may exacerbate both pro‐atherogenic and pro‐thrombotic processes. A study has demonstrated that TMAO enhances platelet recruitment and adhesion through endothelial calcium signaling [15]. In stroke, activated platelets play a dual role, not only contributing to thrombosis but also influencing plaque stability via interactions with immune cells [37]. Under high‐shear conditions at stenotic sites, platelets tend to adhere to damaged endothelium and aggregate [38, 39]. AF is associated with gut dysbiosis [5, 40, 41] and leads to the overproduction of TMAO [42]. Consequently, elevated plasma TMAO levels can accelerate thrombus formation at the site of stenosis, due to platelet hyperreactivity, thereby promoting in situ thrombosis and the adherence of emboli. This concept is reinforced by recent studies demonstrating the association between TMAO and cerebrovascular lesions in patients with AF [18] and the association between peripheral atherosclerosis and AF onset [43]. Shared risk factors, such as age, hypertension, and diabetes mellitus [44, 45] along with common underlying mechanisms, such as inflammation, endothelial dysfunction, and platelet‐mediated thrombosis [46], may partially explain the relationship between atherosclerosis and AF.

Critically, plasma TMAO level was an independent factor that could distinguish between CES and CE as the etiologic subtype of stroke, despite the comparable AF prevalence rates noted between the two groups. This finding underscores the presence of a unique “neuro–cardio nexus” underlying the pathophysiology of CES: the simultaneous presence of AF and stenosis fosters an environment where TMAO‐enhanced platelet reactivity [15] and tissue factor expression [35, 36] may more readily promote thrombosis at stenotic sites, a phenomenon that may be less pronounced in patients with CE who do not have stenosis. Future studies should focus on examining local TMAO levels in thrombi obtained from stenotic segments and correlating these levels with markers of platelet activity. These findings strongly suggest gut microbiota and TMAO as promising therapeutic targets for CES, potentially through dietary modifications, TMAO‐reducing agents, or customized antithrombotic therapies.

This is the first study reporting the presence of a gradient in plasma TMAO levels across stroke subtypes, based on the direct analysis of arterial blood collected proximal to the thrombus, which reflects the thrombotic microenvironment. Previous studies have shown that TMAO crosses the blood–brain barrier and the blood–cerebrospinal fluid barrier [47]. Peripheral venous blood may be influenced by various systemic factors, and the test results may not accurately reflect the true conditions at the site of cerebral vascular lesions.

The present study has several limitations. First, the single‐center study design restricted our ability to draw definitive causal conclusions regarding the relationship between plasma TMAO levels and the specific etiologic subtypes of stroke, underscoring the importance of validation through multicenter prospective cohort studies. Additionally, TMAO levels determined during the acute stroke phase might reflect stress responses rather than baseline metabolic states. Further, we did not assess platelet reactivity or the potential correlations between TMAO levels and stenosis severity, which hampered the mechanistic interpretation of study findings. Finally, the statistical power of subgroup analyses, particularly within the CES group, was constrained by the limited sample size, emphasizing the need for validation studies with larger cohorts to establish the generalizability of our findings and to clarify the pathophysiologic and clinical significance of TMAO across different etiologic subtypes of stroke.

In conclusion, the present study provides novel insights into the differences in plasma TMAO levels observed among patients with stroke due to LAA, CE, and CES, with the highest levels detected in patients with stroke due to CES. Furthermore, plasma TMAO level was an independent predictor differentiating between stroke due to CE and CES. These findings indicate that TMAO, a metabolite generated by the gut microbiota, may serve as a critical link between cardioembolism, gut dysbiosis, and intracranial stenosis. Future large‐scale prospective studies are warranted to further elucidate the role of TMAO in atherosclerosis and thrombosis associated with AF. The present study underscores the potential of targeting gut microbiota and TMAO pathways for the development of personalized prevention and treatment strategies in high‐risk populations.

## Funding

This work was supported by the Construction Project of High‐Level Hospital of Jiangsu Province at The Affiliated Hospital of Xuzhou Medical University (LCZX202414), Research Project on Integrated Management of Chronic Diseases by Traditional Chinese Medicine and Western Medicine of China Science and Technology Development Center for Chinese Medicine (CXZH2024036‐24), and the Xuzhou Municipal Science and Technology Innovation Promotion Project of Xuzhou Science and Technology Bureau (KC22241).

## Ethics Statement

The study protocol was approved by the Ethics Committee of the Affiliated Hospital of Xuzhou Medical University (approval no: XYFY2023‐KL456‐01) and conducted in accordance with the Declaration of Helsinki.

## Consent

Written informed consent was obtained from all participants or their legal representatives before study initiation.

## Conflicts of Interest

The authors declare no conflicts of interest.

## Data Availability

The data that support the findings of this study are available on request from the corresponding author. The data are not publicly available due to privacy or ethical restrictions.
